# Microwave-Assisted Synthesis of a MK2 Inhibitor by Suzuki-Miyaura Coupling for Study in Werner Syndrome Cells

**DOI:** 10.3390/ph8020257

**Published:** 2015-06-03

**Authors:** Mark C. Bagley, Mohammed Baashen, Irina Chuckowree, Jessica E. Dwyer, David Kipling, Terence Davis

**Affiliations:** 1 Department of Chemistry, School of Life Sciences, University of Sussex, Falmer, Brighton, East Sussex, BN1 9QJ, UK; E-Mails: baashen2000@hotmail.com (M.B.); I.Chuckowree@sussex.ac.uk (I.C.); J.E.Dwyer@sussex.ac.uk (J.E.D.); 2 Institute of Cancer and Genetics, Cardiff University School of Medicine, Heath Park, Cardiff, CF14 4XN, UK; E-Mails: KiplingD@cardiff.ac.uk (D.K.); davist2@cardiff.ac.uk (T.D.)

**Keywords:** Suzuki-Miyaura, cross-coupling, MK2 inhibitor, Werner syndrome, cell aging, pyrazoles, heterocycles, microwave-assisted synthesis, boronic acid

## Abstract

Microwave-assisted Suzuki-Miyaura cross-coupling reactions have been employed towards the synthesis of three different MAPKAPK2 (MK2) inhibitors to study accelerated aging in Werner syndrome (WS) cells, including the cross-coupling of a 2-chloroquinoline with a 3-pyridinylboronic acid, the coupling of an aryl bromide with an indolylboronic acid and the reaction of a 3-amino-4-bromopyrazole with 4-carbamoylphenylboronic acid. In all of these processes, the Suzuki-Miyaura reaction was fast and relatively efficient using a palladium catalyst under microwave irradiation. The process was incorporated into a rapid 3-step microwave-assisted method for the synthesis of a MK2 inhibitor involving 3-aminopyrazole formation, pyrazole C-4 bromination using *N*-bromosuccinimide (NBS), and Suzuki-Miyaura cross-coupling of the pyrazolyl bromide with 4-carbamoylphenylboronic acid to give the target 4-arylpyrazole in 35% overall yield, suitable for study in WS cells.

## 1. Introduction

Werner syndrome (WS) is an example of a monogenic segmental progeroid syndrome—a rare human autosomal recessive genetic instability syndrome that mimics many, but not all, of the polygenic features of physiological aging [1,2]. It was first characterized by Dr. Otto Werner [3] and its modern diagnosis often relies upon knowledge of previous family incidence and/or presentation with a series of clinical symptoms, including juvenile bilateral cataracts, scleroderma-like skin and premature graying of the hair [4]. Patients manifest a characteristic premature aging phenotype in their twenties or thirties and exhibit an increased predisposition to a number of pro-inflammatory age-related diseases [1], including type II diabetes mellitus, osteoporosis, and atherosclerosis, with premature death occurring typically from cancer or cardiovascular disease. Patients typically have high levels of inflammatory cytokines and intercellular adhesion molecule-1 (ICAM-1) and the same phenomenon can be observed in cell-based studies, with WS fibroblasts showing pronounced activation of inflammatory signaling pathways [5,6,7]. As a result, WS is often classified as an example of “inflamm-aging” [5,7] to highlight its association with, and patient pre-disposition to, age-related inflammatory disease pathologies [8]. The genetic basis of WS is known, with WS resulting from mutations in the RecQ3 DNA helicase-encoding gene *WRN* that give rise to a pathology attributed for the most part to the key role of RecQ3 in cellular responses to specific types of DNA damage. Lack of RecQ3 leads to frequent DNA replication fork stalling and subsequently genomic instability [9] and WS is classified as a chromosome instability syndrome [10]. The replication fork stalling and an increased pro-oxidant state [11] could provide the trigger for replication stress in WS leading to a decreased ability for WS cells to undergo division [5]. This is supported by the observation that young WS cells show a much reduced division capability compared to cells from normal individuals and resemble fibroblasts that have undergone stress-induced premature senescence [12], which is known from many different stimuli to be transduced by the mitogen activated protein kinase (MAPK) p38 [13,14,15]. This implicates the involvement of p38 signaling in the premature cell cycle arrest in WS and the short replicative life span observed in WS cell cultures. This in turn implicates a reduced ability of WS cells to divide as an underlying cause of accelerated aging in WS [2].

### 1.1. Studies of P38 Inhibitors in WS Cells

**Figure 1:**
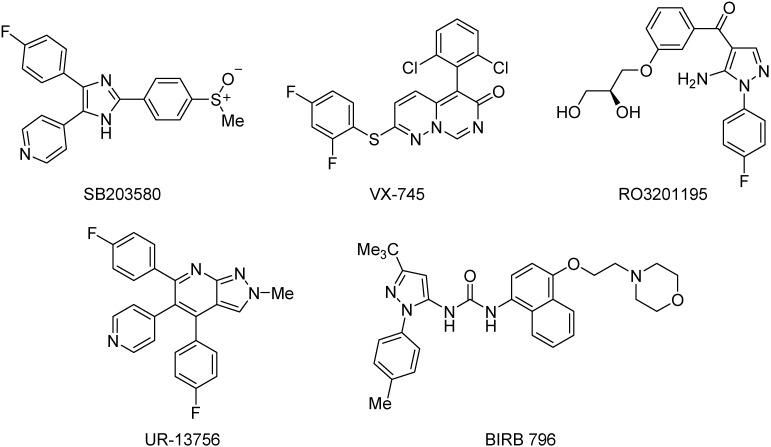
Inhibitors of p38 MAPK examined in WS cells.

The role of stress signaling in Werner syndrome cells has been investigated using a range of small molecule inhibitors (Figure 1) of p38 MAPK [16], including SB203580 [12,17,18], VX-745 [19,20,21], RO3201195 [22,23], UR-13756 [24], and BIRB 796 [25], to study the link between replicative senescence *in vitro* and *in vivo* pathophysiology. WS fibroblasts treated with a p38 inhibitor display an unexpected reversal of the accelerated aging phenotype [12] and, as such, this may provide a suitable model for future therapeutic interventions designed to combat the aging process itself [12,26].

### 1.2. Synthesis and Properties of MK2 Inhibitors

Although progress in the clinical development of successful p38 inhibitors for *in vivo* use is currently a considerable challenge [27], probably frustrated by toxicity issues, it may be possible to elicit a similar biological response by targeting the downstream kinase MAPKAPK2 (MK2) [16,28], which may itself be involved in the phenotypic characteristics seen in WS, including enlarged cellular morphology and prominent F-actin stress fibres [12]. MK2 activity up-regulates the expression of inflammatory pathways [29] and recent data suggest that MK2 acts as a checkpoint kinase that can lead to cell cycle arrest [30]. Furthermore MK2 knock-out mice exhibit normal, healthy phenotypes and are inflammation resistant [31], whereas p38 knock-out mice are lethal [32]. This could indicate that chemical inhibition of MK2 may be less problematic than inhibition of p38, whilst providing similar *in vivo* efficacy.

**Figure 2:**
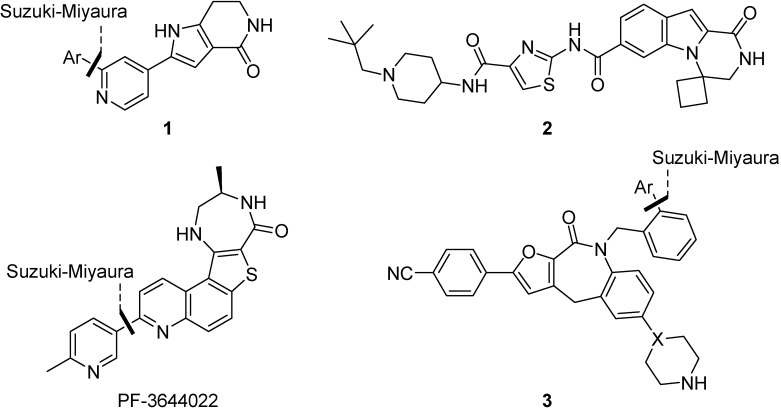
Examples of MK2 inhibitors from different drug discovery programs, showing the use of Suzuki-Miyaura aryl-aryl coupling for late-stage scaffold optimization.

As a consequence of these observations, there has been considerable recent activity in drug discovery programs to develop small molecule inhibitors of MK2 [33]. A number of different chemotypes have been investigated [34], including a series of pyrrolopyridines **1** [35], pyrazinoindolone **2** [36], the benzo[4,5]thieno[3,2-*e*][1,4]diazepinone PF-3644022 [37] and recently tricyclic lactams **3** as a non-ATP-competitive inhibitor scaffold [38], yet issues of selectivity, potency and/or toxicity remain [34,39]. Many, but not all, of these approaches used Suzuki-Miyaura reactions to introduce diversity to an advanced intermediate for scaffold optimization (Figure 2). Since its initial discovery as a Pd-catalyzed stereospecific cross-coupling reaction of 1-alkenylboranes [40], the Suzuki-Miyaura reaction has become one of the most efficient methods for C–C bond formation, in particular for aryl-aryl coupling [41]. It offers many advantages over other methods, in that it is largely unaffected by the presence of water, produces non-toxic inorganic by-products that are easily removed, tolerates a broad range of functional groups and proceeds generally with regio- and stereocontrol [42]. In a recent analysis of reactions used in the pursuit of drug candidates taken from a 2008 data set [43], biaryl linkages were present in 40% of the compounds that were analyzed and the Suzuki-Miyaura reaction occurred in over 4% of all reactions and in 40% of all C–C bond forming reactions. Given the amenability of transition-metal mediated processes to be accelerated under microwave dielectric heating [44], and thus readily automated to probe SAR, it is perhaps not surprising to observe so many uses of this reaction in the development of MK2 inhibitors [28] and such a high occurrence of this reaction in drug discovery programs in general [44].

**Figure 3:**
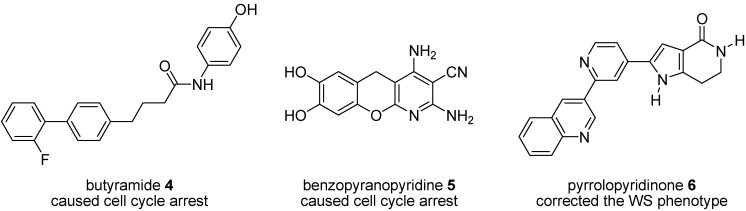
MK2 inhibitor chemotypes and their effect on WS cells.

Given the success of a p38 inhibitor in rescuing accelerated aging in WS cells, we carried out studies on a number of MK2 inhibitors (Figure 3) to establish their effect on cell aging phenotypes using fibroblasts from human WS [28,45,46]. Of these inhibitors, butyramide **4** was reported to act by blocking p38-MK2 docking, thus preventing the activation of MK2 by phospho-p38 [47] but in WS cells caused a stressed morphology and permanent cell cycle arrest [46] by a pathway that appeared not to involve MK2 inhibition [46]. A benzopyranopyridine inhibitor **5** [48] gave a similar cellular response at a concentration that inhibited MK2, but this was thought to be due to non-specific toxicity issues [46]. However, when primary WS fibroblasts were treated with a tetrahydropyrrolo[3,2-*c*]pyridin-4-one inhibitor **6**, the stressed cellular morphology and shortened replicative lifespan were both corrected, although the extension in life span was much smaller than the one seen upon treatment with SB203580 [45]. Given this promising indication, and the wide range of MK2 inhibitors investigated in other cellular models, a rapid route to another MK2 inhibitory chemotype was urgently required to provide added confirmation of the above findings.

## 2. Results and Discussion

**Figure 4:**
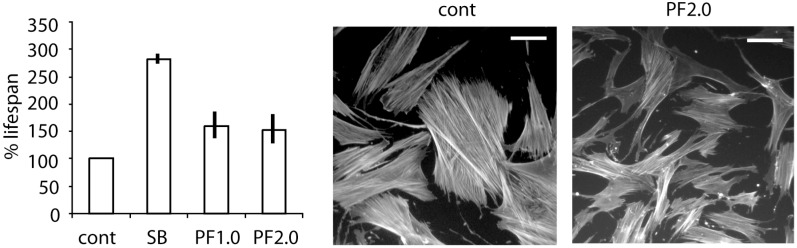
Increased replicative lifespan (±SD; *n* = 3) and improvement cellular morphology in WS cells using PF-3644022. Cont = control, SB = SB203580 at 2.5 µM, PF1.0 and PF2.0 = PF-3644022 at 1.0 and 2.0 µM respectively. White bar = 100 µm (for methods see experimental section).

Our first approach aimed to take advantage of the Suzuki-Miyaura coupling in a rapid route to the Pfizer MK2 inhibitor PF-3644022 [37,49,50]. Although this compound exhibits single digit nanomolar potency against MK2 (IC_50_ 5 nM), cellular activity below 500 nM (U937 TNFα release IC_50_ 150 nM) [50], good selectivity across 200 human kinases and projected human pharmacokinetics sufficient for oral dosing, it was found to lead to acute hepatotoxicity in dogs, probably by disrupting hepatobiliary transporters [39]. Nevertheless, for study in WS cells, its activity and selectivity profile made it a compelling target, with preliminary data using this inhibitor showing extension of cellular replicative capacity in WS fibroblasts and correction of the stressed morphology (Figure 4), a result similar in magnitude to that seen using the tetrahydropyrrolo[3,2-*c*]pyridin-4-one inhibitor **6** [45].

**Figure 5:**
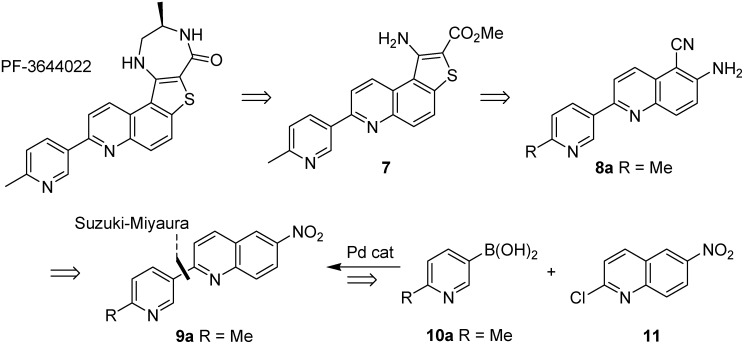
Disconnective approach to the synthesis of PF-3644022, utilizing an early-stage Suzuki-Miyaura cross-coupling reaction.

The development of this inhibitor utilized a late-stage Suzuki-Miyaura coupling to introduce diversity at C-7 of a thieno[3,2-*f*]quinoline ring (see Figure 2) [49], following initial identification of promising SAR studies and cellular potency with a benzo[4,5]thieno[3,2-e][1,4]diazepin-5(2*H*)-one scaffold [50]. Given that our goal was the introduction of a 6-methyl-3-pyridyl substituent at C-7, we set out to incorporate the Suzuki-Miyaura coupling of the pyridinylboronic acid **10a** and 2-chloroquinoline **11** into an early stage of the synthesis (Figure 5). Elaboration of the target inhibitor should then proceed in a similar fashion to Anderson’s route [49]: one-step formation of aminocyanoquinoline **8a** from nitroquinoline **9a**, followed by synthesis of aminobenzothiophene **7** by diazotization and subsequent reaction with methyl thioglycolate before the introduction of the diazepinone ring.

### 2.1. Early-Stage Suzuki-Miyaura Cross-Coupling Reactions for the Synthesis of PF-3644022

**Figure 6:**
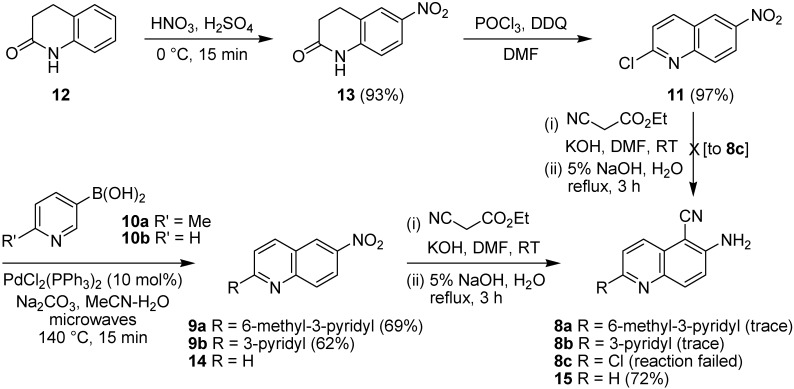
Synthesis of chloroquinoline **11** and subsequent Suzuki-Miyaura cross-coupling.

Starting with a known route to **11** [51], 3,4-dihydroquinolin-2(1*H*)-one (**12**) was nitrated using HNO_3_/H_2_SO_4_ and then treated with phosphorus oxychloride in DMF in the presence of DDQ as oxidant to give 2-chloro-6-nitroquinoline (**11**) in 90% isolated yield over the two steps (Figure 5). Suzuki-Miyaura cross-coupling with 6-methyl-3-pyridinylboronic acid (**10a**) using Na_2_CO_3_ base and PdCl_2_(PPh_3_)_2_ as catalyst in MeCN–H_2_O at 140 °C under microwave irradiation gave the cross-coupled product **9a** in reasonable yield. However, use of the Yamazaki method for the synthesis of *ortho*-aminoaroylnitriles [52], namely treating nitroquinoline **9a** with ethyl cyanoacetate and KOH in DMF at room temperature for up to 72 h, followed by hydrolysis using 5% NaOH (aq) at reflux for 3 h, in accordance with Anderson’s approach, gave only a trace of the 6-amino-5-cyanoquinoline **8a**. Given the wide range of nitroquinolines compatible with this chemistry in Yamazaki’s original report [52], and its previous use in a route to PF-3644022 by Anderson [49], this was unexpected. To probe whether the acidity of the pyridine 6-methyl group had been responsible for this observation, the Suzuki-Miyaura cross-coupling reaction was repeated using 3-pyridinylboronic acid (**10b**), to give the 2-pyridylquinoline **9b** in 62% isolated yield (Figure 6). When this substrate was investigated in the Yamazaki cyanation reaction under identical conditions it behaved in a very similar fashion and only a trace of the cyanoquinoline product **8b** was observed by ^1^H NMR spectroscopic analysis of a complex mixture. Efforts to introduce the Yamazaki cyanation at an earlier stage in the process were similarly unsuccessful. Dihydroquinolone **13** was inert to treatment with ethyl cyanoacetate and KOH in DMF, either at room temperature for 24 h followed by treatment with aqueous base or under more forcing conditions, when heated to 50 °C for 64 h. In both cases only unreacted starting material **13** was obtained. Alternatively, when chloroquinoline **11** was reacted with ethyl cyanoacetate in the presence of base at RT for 64 h (Figure 6) a complex mixture of products was obtained, rather than cyanoquinoline **8c**. It was apparent that either the lactam ring or an electrophilic chloroquinoline function interfered unfavorably with this cyanation reaction. For comparison, Yamazaki cyanation of 6-nitroquinoline (**14**) under identical conditions gave the expected product, 6-amino-5-cyanoquinoline (**15**) in good yield (72%) after basic hydrolysis. Thus it was concluded that an early-stage Suzuki-Miyaura cross-coupling strategy was efficient for biaryl C-C bond formation but the 2-pyridylquinoline products, as well as other quinoline-containing precursors, were incompatible with Yamazaki’s aminoaroylnitrile synthesis and so could not be used for the rapid synthesis of the MK2 inhibitor, PF-3644022.

### 2.2. Suzuki-Miyaura Cross-Coupling Reactions for the Synthesis of (Indolyl)phenylpyrazole MK2 Inhibitor **18**

**Figure 7:**
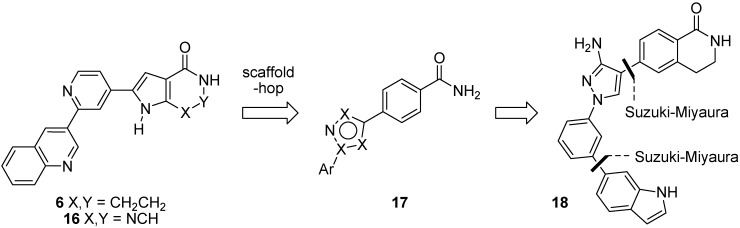
Scaffold-hopping strategy of Velcicky *et al.* [53], showing the use of Suzuki-Miyaura aryl-aryl coupling for scaffold optimization.

Given the success of pyrrolopyridinone **6** in correcting the WS phenotype, we next turned to the scaffold hopping strategy of Velcicky *et al.* [53] for rapid access to a MK2 inhibitor for study in WS cells. Velcicky found that replacing the pyrrolopyrimidinone pharmacophore **16**, which overlays well with pyrrolopyridinone **6**, with a series of benzamide derivatives **17** led to the development of a 3-aminopyrazole **18** that inhibited intracellular phosphorylation of HSP27, as well as LPS-induced TNFα release in cells (Figure 7) [53]. The synthesis of this inhibitor employed two successive Suzuki-Miyaura cross-coupling reactions to establish the biaryl bonds. Given our recent report on the regiocontrolled synthesis of 3-aminopyrazoles [54], this was a compelling scaffold to study.

**Figure 8:**
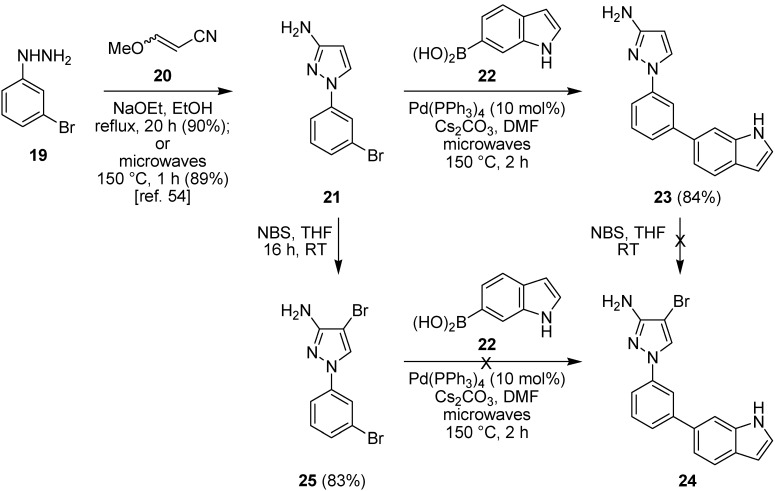
Suzuki-Miyaura cross-coupling for the synthesis of 6-indolyl scaffolds.

The hydrochloride salt of 3-bromophenylhydrazine (**19**) was reacted with 3-methoxyacrylonitrile (**20**) (2 equiv.) in the presence of a large excess of sodium ethoxide in ethanol at reflux for 20 h (Figure 8) to give 3-aminopyrazole **21** in excellent yield (90%), or more rapidly under microwave irradiation at 150 °C for 1 h in a sealed tube to give the same product in comparable yield (89%) [54]. Under the strongly basic conditions, no 5-aminopyrazole regioisomers were isolated or observed. Efforts to improve the microwave-assisted procedure, by switching to *tert*-butyl alcohol in the presence of *tert*-butoxide as base and irradiating at 150 °C for 2 h caused a significant reduction in yield (68%) and so the overnight conductive heating method was adopted as the procedure of choice as it could be readily carried out on gram scale. Suzuki-Miyaura cross-coupling of the arylbromide **21** with 6-indolylboronic acid **22** under microwave irradiation at 150 °C for 2 h in DMF in the presence of cesium carbonate and tetrakis(triphenylphosphine)palladium(0) (10 mol%) gave the coupled product **23** in 84% isolated yield. Unfortunately, bromination at C-4 of aminopyrazole **23** using NBS failed to provide 4-bromopyrazole **24** and instead gave a complex mixture of products, presumably due to competing bromination of the indolyl ring. It was possible to install the C-4 bromo group prior to Suzuki-Miyaura coupling by treating the *N*-(bromophenyl)pyrazole **21** with NBS in THF at RT for 16 h to give dibromide **25** in 83% yield after purification by column chromatography. However, Suzuki-Miyaura cross-coupling of dibromide **25** with the 6-indolylboronic acid **22** under similar conditions did not provide the 4-bromopyrazole **24** and instead gave a complex mixture of products and could not differentiate between the two reactive sites. Although potentially this could be resolved through the use of different halogen moieties, given the difficulties or the need to use protecting group chemistries for the synthesis of the indole-containing inhibitor, we switched to a simpler pyrazole target, benzamide **26**, reported to possess reasonable potency (IC_50_ 2.0 µM), as a more accessible inhibitor for evaluation in WS cells.

### 2.3. Suzuki-Miyaura Cross-Coupling Reactions for the Synthesis of Aminopyrazole MK2 Inhibitor **26**

**Figure 9:**
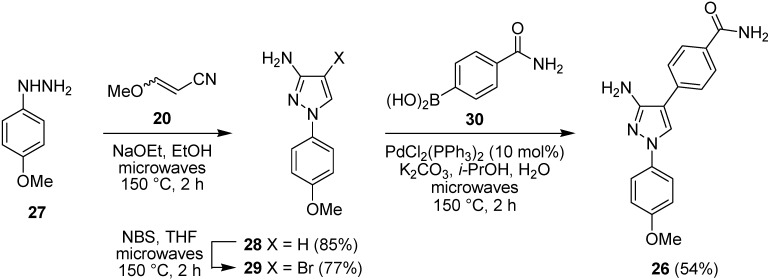
Microwave-assisted synthesis of the MK2 inhibitor **26** using a Suzuki-Miyaura cross-coupling.

Adopting a similar route, the hydrochloride salt of 4-methoxyphenylhydrazine (**27**) was reacted with 3-methoxyacrylonitrile (**20**) (2 equiv.) under basic conditions using sodium ethoxide in ethanol under microwave irradiation at 150 °C for 2 h to give 3-aminopyrazole **28** as the only observed regioisomer in 85% yield (Figure 9). Bromination using NBS in THF under microwave irradiation at 150 °C for 2 h gave the C-4 brominated pyrazole **29** in 77% yield. This rapid microwave-assisted procedure was preferred over a method under ambient conditions, which required stirring with NBS in THF for 16 h but did give bromide **29** in slightly improved yield (82%). Suzuki-Miyaura coupling of the pyrazolylbromide **29** with 4-carbamoylphenylboronic acid (**30**) in *i*PrOH–H_2_O in the presence of potassium carbonate and bis(triphenylphosphine)palladium(II) chloride (10 mol%) under microwave irradiation at 150 °C for 2 h using similar conditions to Velcicky *et al.* [53] gave the coupled product **26** in 54% yield after purification by column chromatography. This route to pyrazole **26** utilized three microwave-assisted steps, each of 2 h duration, starting from commercially-available materials and provided the target MK2 inhibitor in 35% overall yield, suitable for study in WS cells.

### 2.4. Biological Evaluation of Aminopyrazole MK2 Inhibitor **26** in WS Cells

When WS fibroblasts were grown in the presence of inhibitor **26** at 25 µM a small lifespan extension was noted, although it was difficult to ascertain if this was significant, especially as little inhibition of MK2 activity was seen at this concentration [28]. Treatment with inhibitor **26** did, however, show improvement in the cellular morphology of the WS cells at 25 µM and 50 µM. These results were encouraging for the further development of MK2 inhibitors in this and other inflammatory therapeutic areas.

### 2.5. The Role of MK2 in the WS Cell Phenotype and Its Modulation Using Chemical Inhibitors

Effects of MK2 inhibitors on the growth and morphology of WS cells have been ambiguous: inhibitors **4** and **5** resulted in growth inhibition that was not related to MK2 inhibition [46]; inhibitor **6** increased cellular replicative capacity at a concentration that maximally inhibited MK2 [45]; inhibitor **26** showed a small increase in replicative capacity and alleviated the stressed morphology [28]; and finally, PF-3644022 had an effect similar in magnitude to inhibitor **6** at a dose that fully inhibited MK2 (this work). Thus, three MK2 inhibitors with very different chemotypes gave similar results for WS fibroblasts on all cellular parameters tested. These data suggest a role for p38-activated MK2 in replicative senescence in WS fibroblasts, although this role appears to be much reduced compared to that of p38, as the p38 inhibitor SB203580 has much larger effects on the replicative capacity of WS fibroblasts [12]. Possible reasons for the reduced effects of MK2 inhibitors on replicative capacity are that MK2 may be only one of the downstream effectors of p38 responsible for cellular growth arrest [55], or that MK2 inhibitors have a narrow therapeutic window and become toxic to cell growth at levels close to the maximally effective dose [34]. This latter is seen with both PF-3644022 that is effective at 2.0 µM but becomes toxic to cell growth at levels of 5.0 µM and above (unpublished data), and with inhibitor **6** that is maximally effective at 5.0 µM but toxic at 10.0 µM [45]. These data also suggest that MK2 inhibitors may not be as useful therapeutically as p38 inhibitors if the accelerated cell aging seen in WS does underlie the accelerated whole body aging, as suggested [2], but may prove useful in the alleviation of the associated inflammatory conditions that are thought to be MK2 dependent [29]. As inflammatory conditions that may be due to MK2 activation are an increasing issue during the normal human aging process [56], such inhibitors may be of wider therapeutic benefit in the general population. This then provides a good rationale for the continued efforts to find suitable MK2 inhibitors for *in vivo* use.

## 3. Experimental Section

### 3.1. Synthetic Materials and Methods

Commercially available reagents were used without further purification; solvents were dried by standard procedures. Light petroleum refers to the fraction with bp 40–60 °C and ether (Et_2_O) refers to diethyl ether. Column chromatography was carried out using Merck Kieselgel 60 H silica or Matrex silica 60. Analytical thin layer chromatography was carried out using aluminium-backed plates coated with Merck Kieselgel 60 GF_254_ that were visualised under UV light (at 254 and/or 360 nm). Microwave irradiation experiments were performed in a sealed Pyrex tube using a self-tunable CEM Discover or CEM Explorer focused monomodal microwave synthesizer at the given temperature using the instrument’s in-built IR temperature measuring device, by varying the irradiation power (initial power given in parentheses).

Fully characterized compounds were chromatographically homogeneous. Melting points (mp) were determined on a Kofler hot stage apparatus or Stanford Research Systems Optimelt and are uncorrected. Infra–red (IR) spectra were recorded in the range 4000–600 cm^−1^ on a Perkin-Elmer 1600 series FTIR spectrometer using an ATR probe or as a KBr disk (KBr) and are reported in cm^−1^. Nuclear magnetic resonance (NMR) spectra were recorded in CDCl_3_ at 25 °C unless stated otherwise using a Varian VNMRS instrument operating at 400 or 500 MHz or a Bruker DPX 400 or 500 Avance instrument operating at 400 or 500 MHz for ^1^H spectra and 100 or 126 MHz for ^13^C spectra and were reported in ppm; *J* values were recorded in Hz and multiplicities were expressed by the usual conventions (s = singlet, d = doublet, t = triplet, app = apparent, m = multiplet, br = broad). Mass spectra (MS) were determined with a Fisons VG Platform II Quadrupole instrument using atmospheric pressure chemical ionization (APcI), a Waters Q-TOF Ultima using electrospray positive ionization (ES), a Waters LCT premier XE using atmospheric pressure chemical ionization (APcI), an Agilent 6130 single quadrupole with an APcI/electrospray dual source, a Fisons Instrument VG Autospec using electron ionization (EI) at 70 eV (low resolution) or a ThermoQuest Finnigan LCQ DUO electrospray, unless otherwise stated. Some high-resolution mass spectra were obtained courtesy of the EPSRC Mass Spectrometry Service at Swansea, UK using the ionisation methods specified. *In vacuo* refers to evaporation at reduced pressure using a rotary evaporator and diaphragm pump, followed by the removal of trace volatiles using a vacuum (oil) pump.

### 3.2. General Synthetic Procedures

#### General Procedure for Suzuki-Miyaura Cross-Coupling

A solution of 2-chloro-6-nitroquinoline, boronic acid (1.5–1.6 equiv.), aqueous Na_2_CO_3_ solution (2 equiv.) and PdCl_2_(PPh_3_)_2_ (10 mol%) in MeCN (0.2 M) was irradiated at 140 °C for 15 min in a pressure-rated glass tube (35 mL) using a CEM Discover microwave synthesizer by moderating the initial power (200 W). After cooling in a flow of compressed air, the solvent was evaporated *in vacuo* and the residue was triturated with water. Purification by flash column chromatography on SiO_2_ gave the desired 2-pyridinyl-6-nitroquinoline.

### 3.3. Synthetic Experimental Procedures

#### 3.3.1. 3,4-Dihydro-6-nitroquinolin-2(1*H*)-one (**13**)

According to a known literature procedure [51], concentrated sulfuric acid (20 mL) was added to 3,4-dihydro-2-(l*H*)-quinoline (**12**) (1.00 g, 6.79 mmol) in a 100 mL round-bottom flask. The reaction was placed into an ice-acetone bath (−10 °C approx.) and stirred to dissolve the solid. Nitric acid (69%; 0.50 mL, 11.3 mmol, 1.7 equiv.) was added dropwise and the orange-red solution was stirred at −10 to 0 °C for 30 min. The reaction was quenched by pouring onto a stirred ice-water slush (300 mL). The ice was allowed to melt and the precipitate was collected by vacuum filtration, washed with water and air dried under suction to give a beige-coloured solid (1.21 g, 93%), with identical physical and spectroscopic properties to literature data [51].

#### 3.3.2. 2-Chloro-6-nitroquinoline (**11**)

POCl_3_ (1.2 mL, 13.0 mmol) was added dropwise to a stirred solution of **13** (1.00 g, 5.2 mmol) and DDQ (1.30 g, 5.7 mmol) in DMF (20 mL) and the reaction mixture was stirred at RT for 1 h. The reaction mixture was then poured into iced water and the resultant precipitate was collected by vacuum filtration, washed with water and dried in air to give the *title compound* (1.05 g, 97%) as an orange solid, mp 226.8–227.9 °C (lit. [51] mp 235.5–236.5 °C) (Found [ES]: MH^+^, 209.0112. C_9_H_6_ClN_2_O_2_ [*MH*] requires 209.0112); IR (neat) ν*_max_*/cm^−1^ 3068 (C-H), 1619 (C=C), 1523 (NO_2_), 1485 (C=C), 1337 (NO_2_), 1105 (C-N); ^1^H-NMR (500 MHz, *d_6_*-DMSO) δ*_H_*/ppm 9.13 (1H, d, *J* 2.5, 5-CH), 8.78 (1H, d, *J* 9, 4-CH), 8.52 (1H, dd, *J* 9, 2.5, 7-CH), 8.18 (1H, d, *J* 9, 8-CH), 7.84 (1H, d, *J* 9, 3-CH); ^13^C-NMR (126 MHz, *d_6_*-DMSO) δ*_C_*/ppm 153.6 (C), 149.2 (C), 145.3 (C), 141.9 (4-CH), 129.7 (8-CH), 125.9 (C), 125.0 (5-CH), 124.4 (3-CH), 124.1 (7-CH); *m/z* (EI) 208 (M^•+^, 100), 162 (48), 150 (49), 127 (81).

#### 3.3.3. 2-(6-Methylpyridin-3-yl)-6-nitroquinoline (**9a**)

Biaryl **9a** was prepared according to general synthetic procedure 3.2.1 using 2-chloro-6-nitroquinoline (**11**) (0.45 g, 2.20 mmol), 6-methyl-3-pyridinylboronic acid (**10a**) (0.44 g, 3.20 mmol), aqueous Na_2_CO_3_ solution (1.5 M; 3 mL) and PdCl_2_(PPh_3_)_2_ (0.15 g, 0.21 mmol) in MeCN (12 mL). Purification by column chromatography on SiO_2_ gel, gradient eluting with CH_2_Cl_2_ to CH_2_Cl_2_–MeOH (9:1), gave the *title compound* (0.39 g, 69%) as an orange solid, mp 237.5–239.1 °C (Found [ES]: MH^+^, 266.0923. C_15_H_12_N_3_O_2_ [*MH*] requires 266.0924); IR (neat) ν*_max_*/cm^−1^ 3070 (C-H), 2921 (C-H), 1596 (C-C), 1534 (NO_2_), 1327 (NO_2_); ^1^H-NMR (500 MHz, CDCl_3_) δ*_H_*/ppm 9.30 (1H, d, *J* 1, 2'-CH), 8.82 (1H, d, *J* 2, 5-CH), 8.50 (2H, 4'-CH and 7-CH), 8.43 (1H, d, *J* 9, 4-CH), 8.28 (1H, d, *J* 9, 8-CH), 8.05 (1H, d, *J* 9, 3-CH), 7.36 (1H, d, *J* 8, 5'-CH), 2.68 (3H, s, Me); ^13^C-NMR (126 MHz, CDCl_3_) δ*_C_*/ppm 160.6 (C), 158.2 (C), 150.4 (C), 148.4 (2'-CH), 145.5 (C), 138.7 (4-CH), 135.4 (4'-CH), 131.4 (8-CH), 131.3 (C), 126.0 (C), 124.3 (5-CH), 123.4 (7-CH), 123.4 (5'-CH), 120.0 (3-CH), 24.5 (Me).

#### 3.3.4. 2-(Pyridin-3-yl)-6-nitroquinoline (**9b**)

Biaryl **9b** was prepared according to the general procedure 3.2.1 using 2-chloro-6-nitroquinoline (**11**) (0.15 g, 0.71 mmol), 3-pyridinylboronic acid (**10b**) (0.10 g, 1.14 mmol), aqueous Na_2_CO_3_ (1.4 M, 1 mL) and PdCl_2_(PPh_3_)_2_ (0.05 g, 0.07 mmol) in MeCN (3.6 mL). Purification by column chromatography on SiO_2_ gel, eluting with EtOAc–CH_2_Cl_2_ (3:7), gave the *title compound* (0.11 g, 62%) as a yellow solid (Found [ES]: MH^+^, 252.0776. C_14_H_10_N_3_O_2_ [*MH*] requires 252.0767); IR (neat) ν*_max_*/cm^−1^ 3081 (C-H), 1607 (C=C), 1528 (NO_2_), 1337 (NO_2_); ^1^H-NMR (500 MHz, CDCl_3_) δ*_H_*/ppm 9.43 (1H, br s, 2'-CH), 8.84 (1H, d, *J* 2, 5-CH), 8.78 (1H, d, *J* 3, 6'-CH), 8.58 (1H, d, *J* 8, 4'-CH), 8.53 (1H, dd, *J* 9, 2, 7-CH), 8.48 (1H, d, *J* 8, 4-CH), 8.31 (1H, d, *J* 9, 8-CH), 8.08 (1H, d, *J* 9, 3-CH), 7.52 (1H, m, 5'-CH); ^13^C-NMR (126 MHz, CDCl_3_) δ*_C_*/ppm 158.0 (C), 151.2 (6'-CH), 150.4 (C), 149.0 (2'-CH), 138.9 (4-CH), 135.2 (4'-CH), 131.6 (8-CH), 128.5 (C), 128.4 (C), 126.1 (C), 124.3 (5-CH), 123.8 (C), 123.5 (7-CH), 120.2 (3-CH); *m/z* (EI) 251 (M^•+^, 100), 205 (35).

#### 3.3.5. 6-Amino-5-cyanoquinoline (**15**)

6-Nitroquinoline (0.50 g, 2.85 mmol) was added to a solution of ethyl cyanoacetate (0.91 mL, 8.61 mmol) and KOH (0.97 g, 17.0 mmol) in DMF (8.7 mL) and the mixture was stirred for 64 h. The solvent was evaporated *in vacuo* and the residue was dissolved in aqueous NaOH solution (5%; 12 mL) and heated at reflux for 3 h. The reaction mixture was allowed to cool and extracted with CHCl_3_ (3 × 15 mL). The organic extracts were combined, dried (MgSO_4_) and evaporated *in vacuo*. Purification by flash column chromatography on SiO_2_ gel, gradient eluting with CH_2_Cl_2_ to CH_2_Cl_2_–MeOH (9:1), gave the *title compound* (0.35 g, 72%) as an orange solid, mp 181.2–182.8 °C (lit. [57] mp 181 °C) (Found [ES]: MH^+^, 170.0711. C_10_H_8_N_3_ [*MH*] requires 170.0713); IR (neat) ν*_max_*/cm^−1^ 3391 (N-H), 3337 (N-H), 3159 (C-H), 2199 (C≡N), 1635 (N-H), 1615 (C=C), 1337 (C-N); ^1^H-NMR (500 MHz, CDCl_3_) δ*_H_*/ppm 8.74 (1H, d, *J* 3, 2-CH), 8.24 (1H, d, *J* 8, 4-CH), 8.06 (1H, d, *J* 9, 8-CH), 7.47 (1H, m, 3-CH), 7.15 (1H, d, *J* 9, 7-CH), 4.93 (2H, br s, NH_2_); ^13^C-NMR (126 MHz, CDCl_3_) δ*_C_*/ppm 150.3 (6-C), 147.7 (2-CH), 142.4 (C), 136.0 (8-CH), 131.2 (4-CH), 128.9 (C), 123.4 (3-CH), 120.2 (7-CH), 116.1 (CN), 86.9 (5-C).

#### 3.3.6. 3-Amino-1-(3-bromophenyl)-1*H*-pyrazole (**21**)

A solution of 3-bromophenyl hydrazine hydrochloride (1.36 g, 6.00 mmol), 3-methoxyacrylonitrile (1.0 mL, 12.0 mmol) and NaOEt (1.77 g, 26.0 mmol) in dry EtOH (30 mL) was heated at reflux for 20 h. After cooling the mixture to room temperature, water was added and the resultant precipitate was filtered, washed with water and finally dissolved in CH_2_Cl_2_. The organic extract was washed successively with water and brine, dried (MgSO_4_) and evaporated *in vacuo* to give the *title compound* (1.29 g, 90%) as a yellow solid, with identical physical and spectroscopic properties to literature data [54].

#### 3.3.7. 3-Amino-1-[3-(1*H*-indol-6-yl)phenyl]-1*H*-pyrazole (**23**)

A mixture of 3-amino-1-(3-bromophenyl)-1*H*-pyrazole (**21**) (0.20 g, 0.84 mmol), 6-indolylboronic acid (**22**) (0.13 g, 0.25 mmol), caesium carbonate (0.55 g, 1.68 mmol) and Pd(PPh_3_)_4_ (0.10 g, 0.08 mmol) in DMF (4 mL) was irradiated at 150 °C for 2 h in a sealed pressure-rated glass tube (10 mL) using a CEM Discover microwave synthesizer by moderating the initial power (150 W). The mixture was cooled by passing a stream of compressed air through the microwave cavity and water was added (10 mL). The aqueous layer was extracted with EtOAc (3 × 20 mL) and the organic extracts were combined, washed with brine, dried (MgSO_4_) and evaporated *in vacuo*. Purification by column chromatography on silica gel, eluting with hexane–EtOAc (1:1 *v/v*) gave the *title compound* [53] (0.19 g, 84%) as a colorless solid, mp 97–99 °C (Found: M^•+^, 274.1218. C_17_H_14_N_2_ [*M*] requires 274.1222); IR (KBr) ν*_max_*/cm^−1^ 3411 (NH), 2922 (CH), 1626, 1604, 1586, 1479; ^1^H-NMR (400 MHz; *d_6_*-DMSO) δ*_H_*/ppm 11.18 (1H, s, exch. D_2_O, NH), 8.26 (1H, d, *J* 2.6, H-5), 7.96 (1H, m, H-2'), 7.69 (1H, m, H-7''), 7.63 (1H, d, *J* 8.3, H-6'), 7.58 (1H, m, H-5'), 7.46–7.41 (3H, H-4', H-2'' and H-4''), 7.35 (1H, dd, *J* 8.3, 1.6, H-4''), 6.47 (1H, m, H-3''), 5.76 (1H, d, *J* 2.6, H-4), 5.17 (2H, s, exch. D_2_O, NH_2_); ^13^C-NMR (100 MHz, *d_6_*-DMSO) δ*_C_*/ppm 157.5 (C), 134.3 (C), 141.0 (C), 137.0 (C) 133.5 (C), 130.2 (C), 128.5 (CH), 127.9 (CH), 126.8 (CH), 122.7 (CH), 120.9 (CH), 118.8 (CH), 115.2 (CH), 114.9 (CH), 110.0 (CH), 101.5 (CH), 96.7 (CH); *m/z* (EI) 275 (MH^+^, 100%), 274 (M^•+^, 100), 273 (19), 246 (7), 205 (6), 191 (9), 133 (14), 84 (21).

#### 3.3.8. 3-Amino-4-bromo-1-(3-bromophenyl)-1*H*-pyrazole (**25**)

A solution of 3-amino-1-(3-bromophenyl)-1*H*-pyrazole (**21**) (0.16 g, 0.68 mmol) and NBS (0.120 g, 0.68 mmol) in THF (10 mL) was stirred at room temperature for 16 h and then the solvent was vaporated *in vacuo*. The residue was dissolved in EtOAc, filtered through Celite and evaporated *in vacuo*. Purification by column chromatography on silica gel, eluting with hexane–EtOAC (3:1 *v/v*), gave the *title compound* (0.18 g, 83%) as a brown solid, mp 93–95 °C (Found: M^•+^, 314.9005. C_9_H_7_^79^Br_2_N_3_ [*M*] requires 314.9007); IR (KBr) ν*_max_*/cm^−1^ 3415, 3312, 3213, 1629, 1594, 1556; ^1^H-NMR (400 MHz; *d_6_*-DMSO) δ*_H_*/ppm 8.55 (1H, s, H-5), 7.88 (1H, m, H-2'), 7.67 (1H, m, H-5'), 7.37–7.35 (2H, m, H-4' and H-6'), 5.38 (2H, s, exch. D_2_O, NH_2_); ^13^C-NMR (100 MHz, *d_6_*-DMSO) δ*_C_*/ppm 159.9 (C), 142.5 (C), 131.8 (CH), 128.6 (CH), 127.5 (CH), 122.7 (C), 119.3 (CH), 115.1 (CH), 85.2 (C); *m/z* (EI) 275 (MH^+^, 100%); 319 (C_9_H_7_^81^Br_2_N_3_^•+^, 61) 317 (C_9_H_7_^81^Br^79^BrN_3_^•+^, 30), 315 (C_9_H_7_^79^Br_2_N_3_^•+^, 17), 220 (42), 205 (100), 189 (8), 177 (14), 145 (15), 105 (12).

#### 3.3.9. 3-Amino-1-(4-methoxyphenyl)-1*H*-pyrazole (**28**)

The *title compound* was prepared as a yellow solid, exactly in accordance with our previously published microwave-assisted procedure [54].

#### 3.3.10. 3-Amino-4-bromo-1-(4-methoxyphenyl)-1*H*-pyrazole (**29**)

A solution of 3-amino-1-(4-methoxyphenyl)-1*H*-pyrazole (**28**) (0.13 g, 0.68 mmol) and NBS (0.12 g, 0.68 mmol) in THF (4 mL) was irradiated at 150 °C for 2 h in a sealed pressure-rated glass tube (10 mL) using a CEM Discover microwave synthesizer by moderating the initial power (150 W). The mixture was cooled by passing a stream of compressed air through the microwave cavity and evaporated *in vacuo*. The residue was dissolved in EtOAc, filtered through Celite and the solvent was evaporated *in vacuo*. Purification by column chromatography on silica gel, eluting with hexane–EtOAc (3:1 *v/v*), gave the *title compound* (0.14 g, 77%) as a brown solid, mp 93–96 °C (Found: M^•+^, 267.0015. C_10_H_10_^79^BrN_3_O [*M*] requires 267.0007) IR (KBr) ν*_max_*/cm^−1^ 3353 (NH), 1548, 1516, 1403, 1245, 1084, 1041; ^1^H-NMR (400 MHz; *d_6_*-DMSO) δ*_H_*/ppm 8.34 (1H, s, H-5), 7.57 (2H, d, *J* 9.0, H-2' and H-6'), 6.98 (2H, d, *J* 9.0, H-3' and H-5'), 5.15 (2H, s, exch. D_2_O, NH_2_), 3.76 (3H, s, OMe); ^13^C-NMR (100 MHz, *d_6_*-DMSO) δ*_C_*/ppm 157.0 (C), 155.5 (C), 134.2 (C), 127.8 (CH), 118.5 (CH), 114.9 (CH), 95.3 (C), 55.8 (Me); *m/z* (EI) 269 (C_10_H_10_^81^BrN_3_O^•+^, 97), 267 (C_10_H_10_^79^BrN_3_O^•+^, 100).

#### 3.3.11. 3-Amino-4-(4-aminocarbonylphenyl)-1-(4-methoxyphenyl)-1*H*-pyrazole (**26**)

A mixture of 3-amino-4-bromo-1-(4-methoxyphenyl)-1*H*-pyrazole (**29**) (0.15 g, 0.55 mmol), 4-carbamoylphenylboronic acid (**30**) (90 mg, 0.54 mmol), K_2_CO_3_ (0.20 g, 1.44 mmol) and PdCl_2_(PPh_3_)_2_ (40 mg, 0.05 mmol) in *i*PrOH–H_2_O (1:1 *v/v*) (5 mL) was irradiated at 150 °C for 2 h in a sealed pressure-rated glass tube (10 mL) using a CEM Discover microwave synthesizer by moderating the initial power (150 W). The mixture was cooled by passing a stream of compressed air through the microwave cavity and water was added (10 mL). The aqueous layer was extracted with EtOAc (2 × 20 mL) and the organic extracts were combined, washed with brine, dried (MgSO_4_) and evaporated *in vacuo*. Purification by column chromatography on silica gel, eluting with hexane–EtOAc (1:3 *v/v*), gave the *title compound* (90 mg, 54%) as a cream solid, mp 251–253 °C (Found: M^•+^, 308.1281. C_17_H_16_N_4_O_2_ [*M*] requires 308.1273); IR (KBr) ν*_max_*/cm^−1^ 3389 (NH), 1653 (C=O), 1554, 1522, 1399, 1106; ^1^H-NMR (400 MHz; *d_6_*-DMSO) δ*_H_*/ppm 8.59 (1H, s, H-5), 7.96 (1H, br s, exch. D_2_O, N*H*H), 7.90 (2H, d, *J* 8.4, H-2' and H-6'), 7.69 (2H, d, *J* 7.6, H-3'' and H-5''), 7.67 (2H, d, *J* 7.6, H-2'' and H-6''), 7.31 (1H, br s, exch. D_2_O, NH*H*), 7.02 (2H, d, *J* 8.4, H-3' and H-5'), 5.18 (2H, s, exch. D_2_O, NH_2_), 3.51 (3H, s, OMe); ^13^C-NMR (100 MHz, *d_6_*-DMSO) δ*_C_*/ppm 168.2 (C), 158.3 (C), 155.5 (C), 135.1 (C), 133.6 (C), 132.1 (C), 128.9 (CH), 126.5 (CH), 125.5 (CH), 119.5 (CH), 116.7 (CH), 56.0 (Me); *m/z* (EI) 308 (M^•+^, 100).

### 3.4. Biological Procedures

Werner syndrome fibroblast strains AG03141F, AG05229C and AG05229D were obtained from the Coriell Cell Repository (Camden, NJ, USA). All cells were cultured and treated with p38 and MK2 inhibitors and grown to replicative senescence as described previously [45]. The replicative lifespan for inhibitor treated cells is measured as a percentage of the experimental lifespan seen in the respective control (non-inhibitor treated) cells for each cell strain. The results are plotted as mean ± standard deviation. The assay to detect F-actin stress fibres and cellular morphological changes using phalloidin-FITC is exactly as described previously [45].

## 4. Conclusions

Suzuki-Miyaura cross-coupling reactions have been investigated for the synthesis of three different MK2 inhibitors for study in WS cells. Towards the synthesis of PF-3644022, cross-coupling of a 2-chloroquinoline with a 3-pyridinylboronic acid provided the desired biaryl target but the pyridine moiety and the chloroquinoline precursor both proved incompatible with the subsequent Yamazaki cyanation of a 6-nitroquinoline, en route to the benzothiophene ring. Thus, although a relatively early-stage Suzuki-Miyaura coupling would appear incompatible with this cyanation method, alternative strategies for the incorporation of the 3-aminobenzo[*b*]thiophene moiety could be envisaged and thus incorporated with this transformation [58]. Towards the synthesis of a 3-aminopyrazole MK2 inhibitor, bearing a 1-[(6-indolyl)phenyl] substituent, the Suzuki-Miyaura cross-coupling reaction established one biaryl linkage, but the unprotected indole group interfered with the subsequent NBS bromination required to set up the second Suzuki-Miyaura coupling. Use of a simpler 3-aminopyrazole circumvented this problem and provided an MK2 inhibitor for study in WS cells. In all cases with simple aryl bromides, the Suzuki-Miyaura cross-coupling reactions were reliable and relatively efficient. The synthesis of the 3-amino-1-(4-methoxyphenyl)pyrazole MK2 inhibitor proceeded in three steps and 35% overall yield from commercially-available materials, each step complete in 2 h under microwave irradiation. This constitutes an extremely rapid method for access to this chemical tool, of value in elucidating the role of MK2 in accelerated aging in this progeroid syndrome. Although MK2 inhibitors may not be as useful therapeutically as p38 inhibitors, if the accelerated cell aging seen in WS does underlie the accelerated whole body aging, they may prove useful in the alleviation of the associated inflammatory conditions that are thought to be MK2 dependent and have certainly helped to suggest a role for p38-activated MK2 in replicative senescence in WS fibroblasts.
